# Prioritisation of infectious diseases from a public health perspective: a multi-criteria decision analysis study, France, 2024

**DOI:** 10.2807/1560-7917.ES.2024.29.50.2400074

**Published:** 2024-12-12

**Authors:** Dominique Ploin, Mathilde Alexandre, Bruno Ventelou, Didier Che, Bruno Coignard, Nathalie Boulanger, Christophe Burucoa, François Caron, Pierre Gallian, Yves Hansmann, Christian Lienhardt, Philippe Minodier, Henri Partouche, Matthieu Revest, Nadia Saidani, Gilles Salvat, Nicolas Vignier, Sylvie Floreani, Collaborators’ group, Sabine Henry, Bruno Pozzetto, Bruno Hoen, Experts of the criteria weighting group (alphabetically), Ludwig Serge AHO GLELE, Yannick AUJARD, Claude BEAUBESTRE, Sylvia BENZAKEN, Catherine BILGER, Florence BODEAU-LIVINEC, Marc BONNEFOY, Jean-Marc BRIGNON, Linda CAMBON, Pascal CATHEBRAS, Stéphane CHADAPAUD, Marc CHANELIERE, Isabelle CLAUDET-WALKER, Frédérique CLAUDOT, Anne Paule DUARTE, Luc FERRARI, Philippe FRAISSE, Maxime GIGNON, Véronique GILLERON, Emmanuelle GODEAU, Jean-Marie JANUEL, Hatem KALLEL, Fabienne KOCHERT, Rémi LAPORTE, Thomas LEFEVRE, Didier LEPELLETIER, Bénédicte MICHAUD, Isabelle MILLOT, Emmanuel PIEDNOIR, Hugo PILKINGTON, Marion PORCHERIE, Karine POUCHAIN-GREPINET, Thierry PRESTEL, France ROBLOT, Jean-Louis SEVEQUE, Renaud VERDON, Nicole VERNAZZA-LICHT, France WALLET, Experts of the entity rating group (alphabetically), Alexis ARDOIN, Yannick AUFFRET, Hugues AUMAITRE, Pierre BAKHACHE, François BARBIER, Romain BASMACI, Marie-Cécile BAYEUX-DUNGLAS, Anne-Lise BEAUMONT, Samira BENBEKHTI, Agathe BILLETTE de VILLEMEUR, Jordan BIREBENT, Violette BOIVEAU, Donia BOUZID, Camille BREHIN, Sébastien BRUEL, Fabrice BRUNEEL, Fabienne CAHN SELLEM, Michel CARLES, Enrique CASALINO, Bernard CASTAN, Nicolas CAZES, Guillaume CHAPELET, Jean CHASTRE, Bahram CHAYBANY, Yann-Erick CLAESSENS, Hélène COIGNARD, Nived COLLERCANDY, Matthieu COULONGEAT, Etienne de MONTMOLLIN, Nicolas de PROST, Benoit de WAZIERES, Alexis DESCATHA, Jonathan DUCHENNE, Julie DUMOUCHEL, Bernard FABRE-TESTE, Gaëtan GAVAZZI, Jean-François GEHANNO, Serge GILBERG, Xavier GOCKO, Lidvine GODAERT, Marion GRACIET, Hervé HAAS, Sam HRAIECH, Maxime JEAN, Pauline JEANMOUGIN, Stéphanie LARRAMENDY, Josselin le BEL, François-Xavier LESCURE, Charles-Edouard LUYT, Guillaume MARTIN-BLONDEL, Frédéric MECHAI, Vanina MEYSSONNIER, Patrick MIROUX, Emmanuel MONTASSIER, Alex Stéphane NDJIP NDJOCK, Florian NEGRELLO, Ngoc Lam NGUYEN, Mathieu OBERLIN, Céline OCCELLI, Antoine OUZIEL, Juliette PINOT, Julien POISSY, Alain PUTOT, Christophe RAPP, Patrick RAY, Damien ROUX, Léo SAUVAT, David SCHNELL, Jérémie SOMMÉ, Jean-Paul STAHL, Georges THIEBAULT, Franck THOLLOT, Catherine VERDUN-ESQUER, François VIE LE SAGE, Mathieu VIOLEAU, Guillaume VOIRIOT, Michel WOLFF, Experts from Santé publique France who provided the quantitative data (alphabetically), Henriette DE VALK, Florence LOT, Sylvie MAUGAT, Isabelle PARENT.

**Affiliations:** 1 Hospices Civils de Lyon, Hôpital Femme Mère Enfant, Service de Réanimation Pédiatrique et d’Accueil des Urgences, Bron, France; 2 Centre International de Recherche en Infectiologie (CIRI), Laboratoire Vir’Path, Inserm U1111, CNRS UMR5308, ENS de Lyon, Université Claude Bernard - Lyon 1, Lyon, France; 3 Commission spécialisée Maladies Infectieuses et Maladies Émergentes, Haut Conseil de la Santé Publique, Paris, France; 4 Vétérinaires EPICARE, Pertuis, France; 5 Université d’Aix-Marseille, CNRS, AMSE, Marseille, France; 6 Santé publique France, 12 rue du Val d'Osne, Saint-Maurice, France; 7 Université de Strasbourg, Institut de bactériologie, UR3073 PHAVI: groupe Borrelia, Strasbourg, France; 8 Centre National de Référence *Borrelia*, CHRU Strasbourg, Strasbourg, France; 9 CHU de Poitiers, Département des agents infectieux, Laboratoire de bactériologie et hygiène, Poitiers, France, Poitiers, France; 10 Université de Poitiers, Faculté de Médecine et de Pharmacie, Inserm U1070, Poitiers, France; 11 CHU de Rouen, Service des maladies infectieuses, Rouen, France; 12 Université de Rouen Normandie, Université de Caen Normandie, INSERM, DYNAMICURE UMR 1311, Rouen, France; 13 Établissement Français du Sang, Saint Denis, France; 14 Université d’Aix-Marseille-Université de Corse, Unité des Virus Émergents, IRD 190-INSERM 1207, IRBA, Marseille, France; 15 CHU de Strasbourg, Service des maladies infectieuses et tropicales, Strasbourg, France; 16 Université de Montpellier, INSERM, Institut de Recherche pour le Développement, Montpellier, France; 17 Assistance Publique-Hôpitaux de Marseille, Hôpital Nord, Service des urgences pédiatriques, Marseille, France; 18 Université de Paris-Cité, Département de Médecine Générale, Paris, France; 19 CHU de Rennes, Service des maladies infectieuses et réanimation médicale, Rennes, France; 20 Université de Rennes, UMR-1230 BRM (Bacterial RNA and Medicine), Inserm, Rennes, France; 21 Centre hospitalier de Cornouaille, Service de Maladies Infectieuses et Tropicales, Quimper, France; 22 Agence nationale de sécurité sanitaire de l’alimentation, de l’environnement et du travail, ANSES, Maisons-Alfort, France; 23 Hôpitaux universitaires Paris Seine Saint Denis, Hôpital Avicenne, AP-HP, Bobigny, France; 24 Université Paris Cité, Université Sorbonne Paris Nord, UFR SMBH, IAME, INSERM UMR 1137, Bobigny, France; 25 Secrétariat Général, Haut Conseil de la Santé Publique, Paris, France; 26 The members of the Group are listed under Collaborators; 27 CHU de Saint-Etienne, Département des agents infectieux et d'hygiène, Saint-Etienne, France; 28 Centre International de Recherche en Infectiologie (CIRI), Equipe GIMAP, Inserm U1111, CNRS UMR5308, ENS de Lyon, Université Claude Bernard - Lyon 1, Université Jean Monnet de Saint-Etienne, Saint-Etienne, France; 29 Université de Lorraine, École de santé publique - UR 4360 INSPIIRE, Nancy, France; * These authors contributed equally to this work and share last authorship.

**Keywords:** France, epidemiology, Communicable Diseases, epidemiology, Decision Support Techniques, Health Priorities, Humans, Public Health, Surveys and Questionnaires, multi-criteria decision analysis

## Abstract

**Background:**

Within the International Health Regulations framework, the French High Council for Public Health was mandated in 2022 by health authorities to establish a list of priority infectious diseases for public health, surveillance and research in mainland and overseas France.

**Aim:**

Our objective was to establish this list.

**Methods:**

A multi-criteria decision analysis was used, as recommended by the European Centre for Disease Prevention and Control. A list of 95 entities (infectious diseases or groups of these, including the World Health Organization (WHO)-labelled ‘Disease X’) was established by 17 infectious disease experts. Ten criteria were defined to score entities: incidence rate, case fatality rate, potential for emergence and spread, impact on the individual, on society, on socially vulnerable groups, on the healthcare system, and need for new preventive tools, new curative therapies, and surveillance. Each criterion was assigned a relative weight by 77 multidisciplinary experts. For each entity, 98 physicians from various specialties rated each criterion against the entity, using a four-class Likert-type scale; the ratings were converted into numeric values with a nonlinear scale and respectively weighted to calculate the entity score.

**Results:**

Fifteen entities were ranked as high-priorities, including Disease X and 14 known pathologies (e.g. haemorrhagic fevers, various respiratory viral infections, arboviral infections, multidrug-resistant bacterial infections, invasive meningococcal and pneumococcal diseases, prion diseases, rabies, and tuberculosis).

**Conclusion:**

The priority entities agreed with those of the WHO in 2023; almost all were currently covered by the French surveillance and alert system. Repeating this analysis periodically would keep the list updated.

**Key public health message**

**What did you want to address in this study and why?**

As exemplified by the COVID-19 pandemic and large outbreaks of Chikungunya, Ebola virus disease, mpox or Zika virus disease that occurred since the mid-2000s, infectious diseases can present major public health threats. The aim of this study was to identify which infectious diseases should be prioritised in mainland and overseas France in terms of public health, research, and surveillance, and within the context of International Health Regulations.

**What have we learnt from this study?**

We developed 10 criteria for physicians of different specialities to rank 95 infectious diseases or infections. Fifteen were deemed high priority, e.g. the World Health Organization (WHO)-labelled ‘Disease X’, viral haemorrhagic fevers, respiratory viral infections, arboviral (e.g. West Nile, dengue or Zika virus) infections, infections associated with multidrug-resistant bacteria, invasive meningococcal and pneumococcal diseases, prion diseases, rabies, and tuberculosis.

**What are the implications of your findings for public health?**

Our results obtained through a European Centre for Disease Prevention and Control (ECDC) recommended method, support public health planning and emergency preparedness and align with French needs and WHO objectives. They also confirm the applicability of the ECDC approach. Priority diseases found are well covered by the French surveillance and alert system. Repeating the analysis periodically would ensure that the list remains up to date.

## Introduction

Infectious diseases represent a major challenge for public health and emergency preparedness, as recently illustrated by the COVID-19 pandemic (2019−2023) and the outbreaks of Chikungunya (2014), Ebola virus disease (2013 and 2018), Zika virus disease (2016), and mpox (2022) [1]. 

For mainland and overseas France, knowledge on the communicable pathogens that are of current and ongoing public health relevance is key to inform surveillance and research activities. French Overseas Territories are vastly distributed across the globe, and include French Guiana, which borders Brazil and Surinam, as well as islands in the Caribbean Sea and the Indian and Pacific Oceans. While the types of infectious pathogens, as well as the risk that they pose, may vary at local level, an extensive network of air and sea connections across the Overseas Territories, and between them and mainland France, creates potential avenues for introduction of pathogens into places where they were absent before, as well as further spread. Moreover, French Guiana [2,3] and Mayotte [4] are also experiencing considerable migration movements.

Aside from issues related to pathogens’ introduction through maritime and air traffic, more generally, the emergence or re-emergence of infectious diseases followed by dissemination can also potentially pose a threat to human populations. In a constantly evolving world facing severe environmental changes, declining biodiversity [5], and characterised by high people mobility, it is noteworthy that some recent international outbreaks have been caused by vector-borne and zoonotic diseases [1]. In this regard, it is estimated that 60% of infectious diseases are shared between humans and animals, and that 75% of emerging infectious diseases are in fact zoonotic [6]. It is therefore important to identify and monitor pathogens with a One Health perspective [7].

In 2015, the World Health Organization (WHO) issued a list of priority infectious diseases likely to cause a public health emergency [8] with the aim of developing diagnostic tools, treatments, vaccines, as well as surveillance tools of diseases, vectors, and reservoirs, and better prepare healthcare systems for future emergencies.

In this context, the French High Council of Public Health (Haut Conseil de la Santé Publique; HCSP), which is composed of independent public health experts from various fields and which aims to assist France’s leading decision-makers in the field of public health [9], was asked by the French Ministry of Health in October 2022 to draw up a list of priority infectious diseases, for both mainland and overseas France, within the framework of the International Health Regulations [10]. The aim of this study was to establish the French priority list of infectious diseases with a public health perspective.

## Methods

A multidisciplinary Steering Committee, which comprised 17 members of the HCSP’s Expert Committee for Infectious and Emerging Diseases (CS-MiMe), was set up in April 2023 to conduct the study.

### Selecting a methodology to prioritise infectious diseases

To select a methodology for ranking infectious diseases according to public health priority, we first searched in the PubMed database using combinations of the following keywords: ‘infectious diseases’, ‘emerging infectious diseases’, ‘zoonosis’, ‘prioritisation’, ‘disease classification’; restrictions were publication after 1990, and in English or French. The websites of international health organisations (WHO, European Centre for Disease Prevention and Control (ECDC), United States Centers for Disease Control and Prevention (CDC), World Organisation for Animal Health) were also consulted. Based on the review, the Steering Committee selected and endorsed the multi-criteria decision analysis (MCDA) approach to establish the priority list, according to the ECDC methodology [11].

### Overview of the procedure to establish a priority list of infectious entities

**Figure 1:**
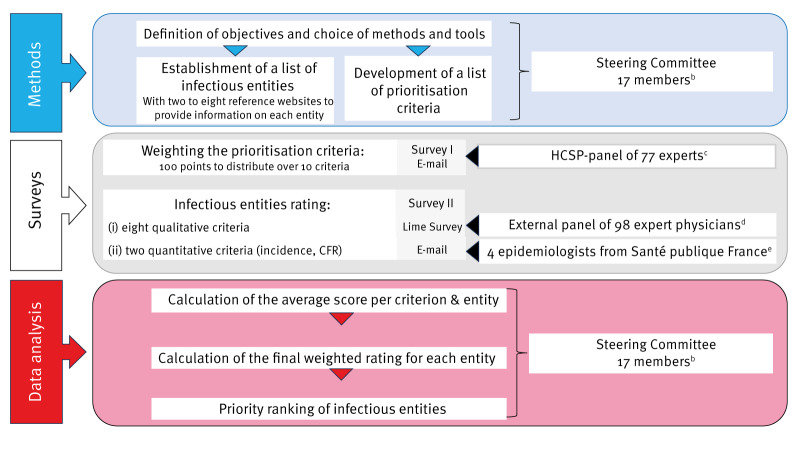
Different phases of a study to establish a list of priority infectious entities^a^ for France and its overseas territories, January−October 2023

HCSP: Haut Conseil de la Santé Publique (French High Council of Public Health); HIV: human immunodeficiency virus.

^a^ An infectious entity is an infectious disease or infectious disease pathogen or group thereof.

^b^ The Steering Committee comprised 17 members from the Expert Committee for Infectious and Emerging Diseases of the HCSP. Two of these members were epidemiologists from the French National Public Health Agency (Santé publique France). Members had experience in the following fields: analysis of the performance of healthcare services and systems, bacteriology, communicable diseases, epidemiology, family medicine, food safety, health economics, HIV, hygiene, infectious and tropical diseases, internal medicine, medical entomology, medical research, paediatrics, parasitology, public health, sexual health, social inequality, veterinary medicine, and virology.

^c^ For the weighting of the 10 criteria, a total of 130 multidisciplinary experts of the HCSP were solicited: 81 participated (62%) and 77 returned valid weightings (59%). The HCSP experts were from the following fields: general practice, health economics, health law, nursing, pharmacy, public health, social sciences and specialised medicine.

^d^ The rating of the entities using the qualitative criteria was performed by external expert physicians; among 169 experts nominated by 14 learned societies, 98 experts from the following specialties participated: emergency medicine, general medicine, geriatrics, infectious diseases, intensive care medicine, occupational health paediatrics, public health (participation rate 58%).

^e^ The rating of these same entities using the quantitative criteria was based on the data provided by four expert epidemiologists from Santé publique France.

Overall, the following steps were applied: (i) establishing a list of infectious diseases to be prioritised (hereafter designated as ‘list of infectious entities’) (ii) developing a list of criteria to prioritise the infectious entities, (iii) assigning weights to the criteria, (iv) rating the criteria for the infectious entities, and finally (v) computing the weighted score on which the ranking of the infectious entities was based (Figure 1). 

### Establishment of a list of infectious entities for prioritisation

To create the list of infectious entities, the Steering Committee considered the lists of notifiable infectious diseases in France as well as the infectious diseases and pathogens subject to a French National Reference Laboratory (NRL). Using the same review process as the one employed to select a methodology to prioritise infectious diseases (see previous sub-section), we also retrieved previously published prioritisation lists. A synthesis of the results of the literature review is presented in Supplementary Table S1 [12-18]. The French reference academic infectious disease textbook (2022 edition [19]) was also reviewed. Pathogen-specific diseases (e.g. tetanus, rabies, malaria), groups of infectious diseases (e.g. bacterial sexually transmitted infections (STIs), infections due to emerging extensively drug-resistant bacteria), and serious clinical forms of infections (invasive bacterial diseases or congenital infections) were all selected as infectious entities. These included strictly human, zoonotic, vector-borne, vaccine-preventable, and tropical diseases (including neglected tropical diseases), whether present or not in mainland France and the overseas territories. The process led to a list of 94 infectious entities, to which was added ‘Disease X’, defined by WHO in February 2018 as ‘the recognition that a serious international epidemic could be caused by an agent not previously known to cause human disease’ [20].

### Development of a list of prioritisation criteria

Based on previously published studies [12-17,21], which the Steering Committee members chose in a consensus manner, 10 criteria (two quantitative (C2 and C3) and eight qualitative (C1 and C4 to C10)) were selected by the committee for the rating of the entities (Table 1).

**Table 1:** List of the criteria to rate infectious entities^a^ and weights assigned to these respective criteria by 77 experts^b^, France, June−August 2023 (n = 10 criteria)

Criterion	Mean weight	Standard deviation
**C1**: Potential for the emergence and spread of the disease or pathogen (e.g. due to environmental changes, globalisation of trade)	12.82	6.54
**C2**: Annual incidence rate (number of cases/100,000 inhabitants)	8.71	4.97
**C3**: Case fatality rate (number of fatal cases/number of cases)	16.09	7.12
**C4**: Individual impact on the patient (e.g. severity of illness, years of life lost and/or loss of quality of life, chronicity, sequelae)	10.36	4.72
**C5:** Societal impact (e.g. due to absenteeism from work or school, excess costs, impact on social cohesion, effects on mental health, level of public concern)	9.65	5.23
**C6**: Impact on the healthcare system (e.g. disorganisation of health services in epidemic situations, impact on prevention, management of other diseases)	12.21	6.84
**C7:** Impact on socially vulnerable populations (e.g. at-risk populations with the possibility of more severe forms and/or delays in treatment), particularly in overseas territories	8.72	4.80
**C8:** Unmet need for prevention (e.g. health education programmes, vaccination, chemoprophylaxis)	7.53	4.12
**C9:** Unmet need for curative treatment (e.g. need for treatment research/development, risk of therapeutic impasse)	8.43	5.23
**C10:** Unmet need in disease surveillance, in mainland and overseas France	5.47	3.13
**Total**	**100**	

^a^ An infectious entity is an infectious disease or infectious disease pathogen or group thereof.

^b^ For the weighting of the 10 criteria, the 130 multidisciplinary experts of the High Council of Public Health (HCSP) were solicited: 81 participated (62%) and 77 returned valid weightings (59%).

### Weighting the prioritisation criteria

The criterion weighting procedure was performed using the Las Vegas method [22]. The 130 expert members of the HCSP were invited to express their appreciation of the relative importance of each criterion by distributing a total number of 100 points to the criteria; each criterion could be assigned 0 to 100 points with the only requirement that the total number of points be equal to 100. The HCSP experts who participated and returned valid (77; 59%) weightings are hereafter referred to as ‘weighters’.

### Infectious entities rating

The rating of the infectious entities using the qualitative criteria was performed by 98 expert physicians (hereafter referred to as ‘raters’) from various medical/biological specialties as described in Supplementary Table S2, nominated by French learned societies, based on their known expertise in the field of infectious diseases and their willingness to participate in this process. Raters were invited by email to take part in the prioritisation process through an online survey. They were requested to rate each entity using the qualitative prioritisation criteria. For appropriate referencing, a list of two to eight weblinks were provided for each entity, presenting fact sheets issued by leading public health and research institutions, including WHO, ECDC, Institut Pasteur, Santé publique France, and the French NRLs.

The rating of the infectious entities using the quantitative criteria (annual incidence rate (C2) and case fatality rate (C3)) was based on the data provided by four expert epidemiologists from Santé publique France.

For qualitative criteria, C1, C4, C5, C6 and C7 had to be rated using a four-class ordinal scale: ‘minimal’, ‘low’, ‘moderate’, ‘high’; and C8, C9, and C10 had to be rated using a four-class Likert-type scale: ‘fully disagree’, ‘partly disagree’, ‘partly agree’, ‘fully agree’.

To optimise participation, the 95 entities were randomly divided into five sets of 19 entities (randomisation by blocks). Each rater was invited to complete at least one set of 19 entities, and if possible, all five. They were informed that only fully completed sets would be considered for analysis. The order of presentation of the sets of entities was randomised; block randomisation was used to achieve 50 ratings per entity. After having completed the first set of entities, raters could opt between stopping their participation or rating a new set of entities, up to five. Those who scored all entities were referred to as complete raters while the others were considered as partial raters.

The online survey was performed using Lime Survey Community Edition 6.3.9 [23]. Its content was tested for acceptability and comprehensibility by 23 physicians.

### Data analysis and scoring

The raters’ responses, collected by the ordinal or Likert-type scales, as well as the quantitative criteria (incidence and case fatality rates) were converted into numeric values in conformity with the nonlinear ECDC quantification scale: 0.005, 0.05, 0.5, and 1 (Table 2) [11]. The final entity score was the weighted sum of the average values assigned by the various experts for each criterion. For *y^j^_i _* the rating of the entity 𝑖 on criterion 𝑗, the final score *Y_i_* is given by:


$$Y_{i}=\sum_{j=1}^{10}w^{j}.y_{i}^{j}$$

**Table 2:** Quantitative and qualitative assessments of infectious entities^a^ by raters, and conversion of these assessments into with ECDC numerical values, France, 2023

Qualitative criteria^b^	Quantitative criteria^c^		Conversion into numeric values according to the nonlinear ECDC quantification scale^d^
Raters’ assessments (ordinal or Likert scale)	Annual incidence rate (n/100,000)	Case fatality rate (%)	
High or fully agree	> 1,000	10–100%	1
Moderate or partly agree	100–1,000	1–10%	0.5
Low or partly disagree	5–100	0.1–1%	0.05
Minimal or fully disagree	< 5	< 0.1%	0.005

ECDC: European Centre for Disease Prevention and Control.

^a^ An infectious entity is an infectious disease or infectious disease pathogen or group thereof.

^b^ For the rating of the entities using the qualitative criteria, an external panel of 169 expert physicians was solicited by 14 learned societies: 98 experts from eight specialties participated (58%).

^c^ Regarding the quantitative criteria, four expert epidemiologists from Santé publique France provided the data.

^d^ The Steering Committee then converted the responses to the ordinal and Likert-type scales and the quantitative data into numeric values using the nonlinear ECDC quantification scale [11].

where *w^j^* are the weights of the different criteria, as determined by the HCSP experts (Table 1). For each entity 𝑖, the prioritisation score *Y_i_* was then on a scale from 0.5 to 100 (the minimum and maximum theoretical values a score could take).

Disease X was analysed separately; incidence was classified as minimal due to its emerging nature whereas case fatality rate was simulated within a range of values using the four possible gradients, enabling Disease X to be positioned with a degree of uncertainty for its rating and ranking.

In addition to MCDA, a single-criterion analysis was conducted. In this analysis, for each criterion *j,* the 10 entities with the highest weighted rating (*w_j_.y^j^_i_*) were selected.

### Quality control analysis

For quality control purposes, three analyses of the rating scores were performed. First, the inter-rater rating homogeneity was analysed by ranking their mean scores on a Z-score scale, and identifying those outliers whose score values differed by more than two standard deviations (SD)s. Second, the consistency of responses between complete and partial raters was examined by comparing their mean scores using a Student’s t-test. Third, inter-rater dissensus was assessed for each entity (including Disease X) through a score dispersion analysis. This was measured by summing up the inter-rater deviations to the mode, weighted by the mode value of each criterion (so that, by construction, a dissensus between minimal and low responses counted less than a dissensus between moderate and high responses, in line with the nonlinear ECDC-quantification scale).

## Results

The prioritisation process was performed on a pre-established list of 95 infectious entities, including Disease X [20], and using eight qualitative and two quantitative pre-selected criteria weighted by an internal panel of 77 experts. The 95 entities were then respectively rated 50 times by a total of 98 expert physicians, each of whom rated an average of 2.6 sets of 19 entities (SD = 1.7).

### Generation of the weight and ratings

For the weighting of the criteria (*w^j^*), the 130 expert members of the HCSP were invited to weight the* j* criteria; 81 (62.3%) members responded and 77 (59.2%) provided a full set of data that were considered valid for analysis. Results of this weighting are shown in Table 1. Incidence and case fatality rates obtained nearly 25% of the total weighting points (8.71 and 16.09). Criteria 7 to 10, which had not been used in former prioritisation studies, obtained nearly 30% of the total weighting points.

For the rating of the entities, two different procedures were used. For the rating of the eight qualitative criteria, 14 learned societies proposed a total of 169 experts; 98 of them participated (58%) to the ratings of 250 sets of 19 entities to obtain 50 ratings of the 95 entities (Supplementary Table S2). For the two quantitative criteria, four epidemiologists from Santé publique France provided the data.

### Quality control analysis

In terms of inter-rater rating homogeneity, no rater score had a Z-score below − 2 SD and four had a Z-score above + 2 SD as illustrated in Supplementary Figure S2. Exclusion of the four outliers would have resulted in the following changes in the high-priority group: − 2 ranks for one entity and  ± 1 rank for four other entities. Without further argument to exclude these data, it was decided to retain the scores of the four experts in the final ranking.

The consistency of responses was analysed between 26 complete raters and 72 partial raters. Although the scores from partial raters tended to be higher than those from complete raters, the difference was not statistically significant (p = 0.51, as shown in Supplementary Figure S3). Subsequently, the responses of complete and partial raters were considered altogether.

Regarding the assessment of inter-rater dissensus (Disease X included), there was a positive correlation between the mode-weighted dissensus indicator and the final entity score; this can be visualised in Supplementary Figure S4. This reflects that the indecision in the rating of certain entities was essentially due to expert hesitation between the moderate (valued at 0.5) and high (valued at 1) categories.

### Distribution of ratings and ranking of the 95 entities

The score of Disease X ranged between 59.4 and 75.4, according to the case fatality ratio simulation, which would end-up in Disease X being on first or second rank. As shown in Supplementary Figure S5, entities with a score above 40, including Disease X, can be isolated; they were grouped together into a set of 15 high-priority entities. The 32 entities with a score between 25 and 39 were classified in a set of low-priority entities. The remaining 48 entities with a score under 25 were classified as non-priority entities (Supplementary Figure S5). The ranking of the 95 entities and the categorisations of priority are summarised in Table 3. Forty-three of the 46 prioritised entities (excluding Disease X) (Table 3) were currently covered by the French surveillance and alert system (Supplementary Table S3).

**Table 3:** Results of ranking infectious disease entities from a public health perspective using a multi-criteria decision analysis and categorisation of priority, France, 2023−2024 (n = 95 entities)

Rank	Name of the entity (score)
**High-priority group: score > 40**	
**0.**	Disease X **(59.4 to 75.4)**
**1.**	Viral haemorrhagic fevers^a^ **(62.9)**
**2.**	ARIs due to viruses other than influenza, emerging coronaviruses, RSV and hMPV^b^ **(56.0)**
**3.**	Mosquito-borne arboviruses^c^ **(55.7)**
**4.**	Influenza virus infections with zoonotic potential **(55.2)**
**5.**	Seasonal influenza A and B **(53.7)**
**6.**	Diseases due to infections with emerging coronaviruses (SARS, MERS, COVID-19) **(49.3)**
**7.**	RSV and hMPV respiratory infections **(48.6)**
**8.**	Creutzfeldt–Jakob disease and other human TSEs **(48.0)**
**9.**	Systemic infections due to MDR bacteria^d^ **(46.9)**
**10.**	Infections due to emerging XDR bacteria **(45.5)**
**11.**	Invasive infections due to *Neisseria meningitidis* **(44.82)**
**12.**	Rabies **(44.3)**
**13.**	Tuberculosis due to antibiotic-susceptible strains **(43.7)**
**14.**	Invasive pneumococcal disease **(43.3)**
**Low-priority group: 25 < score < 40**	
**15.**	Plague **(39.8)**
**16.**	Invasive yeast and filamentous fungal infections (e.g. *Candida*, *Aspergillus*) **(39.6)**
**17.**	Invasive infections due to *Enterobacterales* **(38.7)**
**18.**	Rotavirus gastroenteritis **(37.9)**
**19.**	Drug-resistant tuberculosis^e^ **(37.2)**
**20.**	Tetanus **(37.2)**
**21.**	Viral gastroenteritis excluding rotavirus **(37.1)**
**22.**	Listeriosis **(36.9)**
**23.**	Invasive infections due to *Staphylococcus aureus* **(36.8)**
**24.**	Invasive tropical mycoses^f^ (**36.1)**
**25.**	Melioidosis **(36.0)**
**26.**	Measles **(34.9)**
**27.**	Cutaneous infections of aquatic origin^g^ **(33.6)**
**28.**	HIV infection **(32.8)**
**29.**	Food-borne gastroenteritis/food poisoning^h^ **(32.1)**
**30.**	Severe viral infections**^i^** in immunocompromised patients **(32.0)**
**31.**	Ectoparasitoses including scabies, pediculosis and bed bug infestation **(31.84)**
**32.**	Bacterial sexually transmitted infections^j^ **(31.76)**
**33.**	Cancers and other diseases caused by human papillomaviruses **(30.8)**
**34.**	Orthopoxvirus infections including those causing smallpox and mpox **(30.63)**
**35.**	Invasive infections due to *Streptococcus pyogenes* and other invasive streptococci (*S. suis, S. dysgalactiae*) **(30.3)**
**36.**	Enterovirus infections excluding those causing poliomyelitis **(30.1)**
**37.**	Botulism **(30.0)**
**38.**	Legionellosis **(29.7)**
**39.**	Tick-borne encephalitis **(28.0)**
**40.**	Haemolytic uraemic syndrome **(27.5)**
**41.**	Diphtheria **(27.3)**
**42.**	*Clostridioides difficile* infections **(26.9)**
**43.**	Malaria **(26.5)**
**44.**	Nocardiosis **(25.8)**
**45.**	Cholera **(25.6)**
**46.**	Infections due to hypervirulent clonal strains of *Klebsiella pneumoniae* **(25.2)**
**Non-priority group: score < 25**	
**47.**	Invasive infections due to coagulase-negative staphylococci **(24.5)**
**48.**	Invasive infections due to *Haemophilus influenzae* b **(24.4)**
**49.**	Poliomyelitis **(24.1)**
**50.**	Diseases induced by *Helicobacter pylori* **(23.9)**
**51.**	Cancers induced by and severe infections due to HHV 8 **(23.81)**
**52.**	Cancers induced by Epstein−Barr virus **(23.78)**
**53.**	Diseases induced by HTLV types 1 and 2^k^ **(23.3)**
**54.**	Congenital cytomegalovirus infection **(23.1)**
**55.**	Gastroenteritis and parasitic enterocolitis^l^ **(22.4)**
**56.**	Hepatitis B/hepatitis D **(22.8)**
**57.**	Anthrax **(22.4)**
**58.**	Systemic enterococcal infections (by *Enterococcus faecalis* and *E. faecium*) **(21.2)**
**59.**	Hepatitis C **(20.8)**
**60.**	Mycobacterioses (excluding tuberculosis and leprosy) **(20.7)**
**61.**	Leprosy **(20.1)**
**62.**	Leptospirosis **(20.1)**
**63.**	Chagas disease **(19.8)**
**64.**	Pneumocystis pneumonia **(19.4)**
**65.**	Whooping cough **(19.19)**
**66.**	Atypical pneumonia^m^ **(19.17)**
**67.**	Congenital rubella **(18.9)**
**68.**	Haemorrhagic fever with renal syndrome due to hantaviruses (e.g. Dobrova, Puumala and Seoul viruses) **(18.89)**
**69.**	Urogenital/intestinal schistosomiasis **(18.75)**
**70.**	Cutaneous or visceral leishmaniases **(18.6)**
**71.**	Hepatitis E **(18.3)**
**72.**	Lyme disease **(18.2)**
**73.**	Typhoid and paratyphoid fevers **(17.8)**
**74.**	Cystic and alveolar echinococcosis **(17.43)**
**75.**	Parvovirus B19 infections **(17.41)**
**76.**	Varicella-zoster virus infections **(17.39)**
**77.**	Congenital toxoplasmosis **(17.3)**
**78.**	Severe herpes simplex virus types 1/2 infections **(16.7)**
**79.**	Q fever **(16.6)**
**80.**	Rickettsioses **(15.93)**
**81.**	Systemic *Streptococcus agalactiae* infections **(15.91)**
**82.**	Hepatitis A **(15.20)**
**83*.***	Dermatophytoses (caused by *Microsporum* and *Trichophyton*) **(15.16)**
**84.**	Intestinal nematodiases (**14.4)**
**85.**	Anaplasmosis and other tick-borne bacterial infections (such as infections with *Ehrlichia*) (**14.0)**
**86.**	Whipple's disease **(13.0)**
**87.**	Filariases, cutaneous and visceral *larva migrans* **(12.6**)
**88.**	Tularaemia **(12.4)**
**89.**	Mumps **(12.0)**
**90.**	Bartonellosis **(11.8)**
**91.**	Brucellosis **(11.2)**
**92.**	Bacterial inoculation diseases^n^ (e.g. Erysipeloid, rat-bite fever) **(11.0)**
**93.**	Distomatoses (**10.9)**
**94.**	Pasteurellosis **(9.9)**

ARIs: acute respiratory infections; HHV 8: human herpesvirus type 8; HIV: human immunodeficiency virus; hMPV: human metapneumovirus; HTLV: human T-Lymphocytic virus; MDR: multidrug resistant; MERS: Middle East respiratory syndrome; RSV: respiratory syncytial virus; SARS: severe acute respiratory syndrome; TSEs: transmissible spongiform encephalopathies; XDR: extensively drug-resistant.

^a^ Crimean−Congo haemorrhagic fever, Ebola, Lassa fever, infections with New World arenaviruses, Marburg virus disease, Nipah virus infection, Hendra virus infections, Omsk haemorrhagic fever.

^b^ Enterovirus, human rhinoviruses A to C, parainfluenza viruses 1 to 4, seasonal coronaviruses.

^c^ Chikungunya virus, dengue virus, Japanese encephalitis virus, Rift Valley fever virus, West Nile virus, yellow fever virus, Zika virus.

^d^ *Acinetobacter baumannii*, *Burkholderia cepacia*, *Pseudomonas aeruginosa*.

^e^ Drug-resistant tuberculosis encompasses MDR-TB (strains that are resistant to at least both rifampicin and isoniazid) and XDR-TB (strains that are resistant to rifampicin (and possibly isoniazid) and at least one fluoroquinolone (moxifloxacin or levofloxacin) and at least one of the other two group A drugs (bedaquiline or linezolid) [37].

^f^ Due to *Blastomyces*, *Coccidioides*, *Cryptococcus, Histoplasma*.

^g^ Due to *Aeromonas, Mycobacterium marinum,* *Vibrio vulnificus* or *Shewanella.*

^h^ Due to *Arcobacter*, *Bacillus cereus*, *Campylobacter*, *Clostridium perfringens*, *Escherichia coli*, non-typhoidal *Salmonella*, *Shigella*, *Vibrio parahaemolyticus or Yersinia*.

^I^ Adenovirus, cytomegalovirus, Epstein–Barr virus or human polyomavirus infections. Severe infections were those causing for example encephalitis, interstitial pneumonia, colitis or hepatitis.

^j^ *Chlamydia trachomatis* infection, gonococcal infections, *Mycoplasma genitalium* infection or *Treponema pallidum* infection.

^k^ Adult T leukaemia/lymphoma and tropical spastic paraparesis.

^l^ Due to *Cryptosporidium*, *Cyclospora*, *Entamoeba histolytica* or *Giardia*.

^m^ Due to *Chlamydia pneumoniae* or *C. psitacci or Mycoplasma pneumoniae.*

^n^ 'Inoculation diseases' refers to zoonotic diseases caused by inoculation (i.e. by transmission of pathogens through animal bites/scratches/stings).

Disease X was mentioned as rank 0; its rating score is provided as an interval given the simulation of the case fatality rate.

### Contribution of the different criteria on the scores of infectious entities

**Figure 2:**
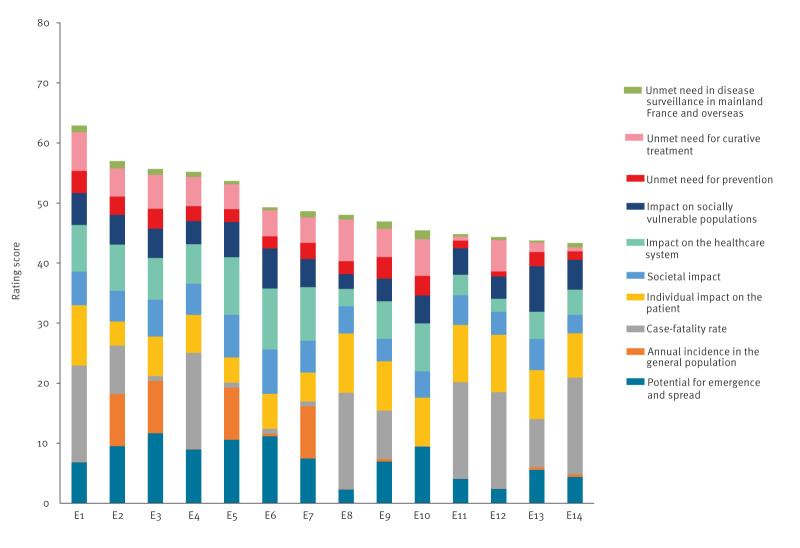
Contribution of the different prioritisation criteria on the scores of high-priority infectious entities, France, 2024 (n = 14 entities)

Supplementary Table S4 shows the respective contribution (*w_j_*.*y^j^_i_*) of the different criteria on the scores of the entities, both in all 94 entities and in the 14 high-priority entities, i.e. excluding Disease X. Whereas the individual impact on the patient criterion had the highest contribution (20.4%) when considering the 94 entities, case fatality rate had the highest contribution (17.7%) in the 14 high-priority entities, followed by the criteria ‘individual impact on the patient’ (14.7%), ‘potential for emergence’ (14.5%), and ‘impact on the healthcare system’ (12.8%). As shown in Figure 2 for high-priority entities, case fatality rate had the highest contribution for viral haemorrhagic fevers, influenza infections with zoonotic potential, prion disease, invasive meningococcal and pneumococcal diseases, and rabies.

hMPV: human metapneumovirus; MDR: multidrug resistant; RSV: respiratory syncytial virus; XDR: extensively drug-resistant.

E1: viral haemorrhagic fevers; E2: acute respiratory infections due to viruses other than influenza, emerging coronaviruses, RSV and hMPV; E3: mosquito-borne arboviruses; E4: influenza virus infections with zoonotic potential; E5: seasonal influenza A and B; E6: diseases due to infections with emerging coronaviruses (severe acute respiratory syndrome, Middle-East respiratory syndrome, COVID-19); E7: RSV and hMPV respiratory infections; E8: Creutzfeldt–Jakob disease and other human transmissible spongiform encephalopathies; E9: systemic infections due to MDR bacteria; E10: infections due to emerging XDR bacteria; E11: invasive infections due to *Neisseria meningitidis*; E12: rabies; E13: tuberculosis due to antibiotic-susceptible strains; E14: invasive pneumococcal disease.

### Top 10 entities according to single-criterion analysis

In addition to MCDA, we considered each of the eight qualitative criteria one at a time, and for each criterion (*j*) we ranked the entities (*i*) according to their weighted rating (*w_j_*.*y^j^_i_*) for that criterion. The 10 entities with the highest weighted rating for each criterion were selected (Table 4). In addition to the 14 entities already ranked as high-priority by MCDA, entities prioritised using this approach included: drug-resistant tuberculosis, HIV infection, measles, and ectoparasitoses for their impact on vulnerable populations; ectoparasitoses, aquatic skin infections, Chagas disease, anaplasmosis and other tick-borne bacterial infections, human T-Lymphocytic virus (HTLV) type 1 or type 2-induced diseases, severe viral infections in the immunocompromised, dermatophytosis, and parvovirus B19 infections for infections with unmet need in disease surveillance, particularly in overseas territories; congenital cytomegalovirus (CMV) infection, severe Epstein−Barr virus (EBV) infections and tick-borne encephalitis for infections with unmet need for prevention; tetanus, poliomyelitis, drug-resistant tuberculosis, and haemolytic uraemic syndrome for their individual impact on patients; bacterial STIs and measles for their potential to emerge or spread; viral gastroenteritis for its impact on the healthcare system; and tick-borne encephalitis and HTLV type 1 or type 2-induced diseases for infections with no available curative treatment.

**Table 4:** Result of single-criterion analysis, with the ranking of the top 10 entities according to each qualitative criterion, France, 2024 (n = 8 qualitative criteria)

Rank	Potential for the emergence and spread of the disease or pathogen	Individual impact on the patient	Societal impact	Impact on the healthcare system	Impact on socially- vulnerable populations	Unmet need for prevention	Unmet need for curative treatment	Unmet need in disease surveillance in mainland and overseas France
**1^st^**	Mosquito-borne arboviruses^a^	Viral haemorrhagic fevers^b^	Disease X	Seasonal influenza A and B	Tuberculosis due to antibiotic-susceptible strains	Disease X	Creutzfeldt–Jakob disease and other human TSEs	Disease X
**2^nd^**	Diseases due to infections with emerging coronaviruses (SARS, MERS, COVID-19)	Creutzfeldt–Jakob disease and other human TSEs	Diseases due to infections with emerging coronaviruses (SARS, MERS, COVID-19)	RSV and hMPV respiratory infections	**Drug-resistant tuberculosis^c^**	Viral haemorrhagic fevers^b^	Disease X	**Ectoparasitoses including scabies, pediculosis and bed bug infestation**
**3^rd^**	Seasonal influenza A and B	Rabies	Seasonal influenza A and B	Infections due to emerging XDR bacteria	Disease X	Systemic infections due to MDR bacteria^d^	Viral haemorrhagic fevers^b^	**Cutaneous infections of aquatic origin^e^**
**4^th^**	Disease X	Invasive infections due to *Neisseria meningitidis*	RSV and hMPV respiratory infections	Viral haemorrhagic fevers^b^	Diseases due to infections with emerging coronaviruses (SARS, MERS, COVID-19)	Mosquito-borne arboviruses^a^	Infections due to emerging XDR bacteria	**Chagas disease**
**5^th^**	Acute respiratory infections due to viruses other than influenza, emerging coronaviruses, RSV and hMPV	**Tetanus**	Infections due to emerging XDR bacteria	Acute respiratory infections due to viruses other than influenza, emerging coronaviruses, RSV and hMPV	**HIV infection**	Infections due to emerging XDR bacteria	Mosquito-borne arboviruses^a^	**Anaplasmosis and other tick-borne bacterial infections**
**6^th^**	Infections due to emerging XDR bacteria	**Poliomyelitis**	Viral haemorrhagic fevers^b^	Mosquito-borne arboviruses^a^	**Measles**	**Congenital cytomegalovirus infection**	Rabies	Infections due to emerging XDR bacteria
**7^th^**	Influenza virus infections with zoonotic potential	Systemic infections due to MDR bacteria^d^	Acute respiratory infections due to viruses other than influenza, emerging coronaviruses, RSV and hMPV	Influenza virus infections with zoonotic potential	Seasonal influenza A and B	Acute respiratory infections due to viruses other than influenza, emerging coronaviruses, RSV and hMPV	**Tick-borne encephalitis**	**Diseases induced by human T-Lymphocytic virus types 1–2^f^**
**8^th^**	**Bacterial sexually transmitted infections^g^**	**Drug-resistant tuberculosis^c^**	Mosquito-borne arboviruses^a^	Systemic infections due to MDR bacteria^d^	**Ectoparasitoses including scabies, pediculosis and bed bug infestation**	RSV and hMPV respiratory infections	**Diseases induced by Human T-Lymphocytic virus types 1–2^f^**	**Severe viral infections^h^ in immunocompromised patients**
**9^th^**	**Measles**	**Haemolytic uraemic syndrome**	Influenza virus infections with zoonotic potential	**Rotavirus gastroenteritis**	Viral haemorrhagic fevers^b^	**Tick-borne encephalitis**	Influenza virus infections with zoonotic potential	**Dermatophytoses (caused by *Microsporum* and *Trichophyton*)**
**10^th^**	RSV and hMPV respiratory infections	Tuberculosis due to antibiotic-susceptible strains	Systemic infections due to MDR-bacteria^d^	**Viral gastroenteritis excluding rotavirus**	Acute respiratory infections due to viruses other than influenza, emerging coronaviruses, RSV and hMPV	**Cancers induced by Epstein−Barr virus**	Infections due to emerging XDR bacteria	**Parvovirus B19 infections**

TSE: transmissible spongiform encephalopathy; hMPV: human metapneumovirus; MDR: multidrug resistant; RSV: respiratory syncytial virus; XDR: extensively drug-resistant.

^a^ Chikungunya virus, dengue virus, Japanese encephalitis virus, Rift Valley fever virus, West Nile virus, yellow fever virus, Zika virus.

^b^ Crimean-Congo haemorrhagic fever, Ebola, Lassa fever, infections with New World arenaviruses, Marburg virus disease, Nipah virus infection, Hendra virus infections, Omsk haemorrhagic fever.

^c^ Drug-resistant tuberculosis encompasses MDR-TB (strains that are resistant to at least both rifampicin and isoniazid) and XDR-TB (strains that are resistant to rifampicin (and possibly isoniazid) and at least one fluoroquinolone (moxifloxacin or levofloxacin) and at least one of the other two group A drugs (bedaquiline or linezolid) [37].

^d^ *Acinetobacter baumannii*, *Burkholderia cepacia*, *Pseudomonas aeruginosa*.

^e^ Due to *Aeromonas, Mycobacterium marinum,* *Vibrio vulnificus* or *Shewanella*.

^f^ Adult T leukaemia/lymphoma and tropical spastic paraparesis.

^g^ *Chlamydia trachomatis* infection, gonococcal infections, *Mycoplasma genitalium* infection or *Treponema pallidum* infection.

^h^ Adenovirus, cytomegalovirus, Epstein–Barr virus or human polyomavirus infections.

The entities NOT previously identified in the high-priority category using multi-criteria decision analysis are displayed in bold font.

## Discussion

In order to achieve objectivity, transparency, and reproducibility, the present study complied with the requirements of the MDCA method recommended by the ECDC by (i) submitting a wide range of diseases (i.e. infectious entities) for the prioritisation process; (ii) defining a reasonable number of non-redundant and explicit criteria, both qualitative (n = 8) and quantitative (n = 2) to assess the diseases’ relative public health importance, and using four-class ordinal and Likert-type scales for rating the diseases according to the criteria; (iii) weighting criteria by a panel of HCSP experts with public health expertise; (iv) rating infectious entities by a panel of 98 raters from various specialties using block randomisation, allowing each entity to be rated 50 times; and (v) highlighting entities that were assigned a high score using a nonlinear scale for each criterion.

As in previous studies [17,24-31], the present analysis confirms the value of the ECDC-recommended MCDA method for disease prioritisation. The use of novel approaches for the selection of entities, the selection of criteria, the choice of experts to weight the criteria, the choice of raters, and the use of the Lime Survey tool to carry out the ratings, together with the single- and multi-criteria rating of the entities, enabled to achieve our goals within 6 months, while most international studies have been conducted over longer time spans [13-15,32]. Using such a robust methodology, allowed by a high number of raters and a limited number of qualitative criteria, one could consider repeating this prioritisation exercise periodically (i.e. every 3–4 years), as recommended by the WHO [33] to consider newly emerging diseases and the change in priorities over time.

Despite the above-mentioned efforts, one could not avoid a certain level of subjectivity in the rating of qualitative criteria, which is inevitable in these types of surveys. In addition, the WHO prioritisation list may have influenced the scoring but there is no indicator able to measure such influence. Limiting the number of qualitative criteria in comparison to the number of quantitative ones and selecting clinical experts experienced in the field of infectious diseases contribute to minimise this bias. It should also be noted that many study participants had worked both in mainland and overseas France, with several having public health expertise on certain Overseas Territories (data not shown). Importantly, the mean scores between partial and complete raters were not significantly different, highlighting a lack of training effect for those who assessed all 95 entities. Another limitation is the categorisation of the 95 entities into three priority levels (‘high-priority’, ‘low-priority’, and ‘non-priority’). The thresholds used for this categorisation could not be defined a priori since no previous study had used the criteria proposed herein. The decision of considering scores of 40 and 25 as thresholds was not based on statistical analysis, and the boundaries we considered to delineate the three groups are probably debatable. However, this categorisation method, also proposed by Balabanova et al. [14] and Klamer et al. [17], provides a ‘macro-hierarchy’ that is easy to use for educational or practical purposes. An additional limitation is the arbitrary attribution of an incidence class to groups of entities with variable incidence rates according to the epidemic context, including the geographical location (e.g. the incidence of dengue is very different whether in mainland France or in tropical overseas territories). This bias was mitigated by aligning the incidence with that of the most frequent disease among a group of diseases (i.e. chlamydiosis among bacterial STIs), and, in case of geographical disparities, with that of the French territory with the highest incidence (i.e. French Caribbean islands for dengue).

Interestingly, the present results are aligned with the WHO prioritisation list, which mainly relied on international epidemiological criteria [20] and included among others, emerging arbovirus diseases, respiratory viruses, infections caused by MDR bacteria, and invasive meningococcal and pneumococcal diseases. In our study, the high level of prioritisation of some entities, such as viral haemorrhagic fevers, mosquito-borne arboviruses, influenza virus infections with zoonotic potential or diseases due to emerging coronaviruses is in line with the One health approach advocated by WHO, which promotes a better understanding of the zoonotic reservoirs and vectors implicated in the spread of new infectious diseases [6].

The priorities revealed through the current work consider relevant criteria such as, for example, the concept of emergence, and the impact on society and the healthcare system − although the impact on patients was ultimately the preferred criterion. Remarkably, four criteria had not previously been used in former prioritisation studies. These consisted of the unmet need in disease surveillance and notably the impact on vulnerable populations both in mainland France and overseas territories, as well as the unmet needs for prevention and curative treatments. Together, these four criteria obtained nearly 30% of the weighting points. Because they constitute important public health objectives, their inclusion is worth considering in future prioritisation work.

Quite unexpectedly, non-influenza respiratory viruses (seasonal coronaviruses, entero-rhinoviruses, parainfluenza viruses) ranked second, with a score of 56.0, probably because of the burden they represent at both ends of life, but also because of the emergence potential of certain viruses, such as Nipah and Hendra viruses in the *Paramyxoviridae* family. Infections caused by influenza viruses with zoonotic potential ranked fourth (score of 55.2), followed by seasonal influenza caused by influenza A and B viruses (score of 53.7), infections by emerging coronaviruses (SARS-CoV-1, MERS-CoV, SARS-CoV-2) (score of 49.3) and respiratory infections caused by respiratory syncytial virus (RSV) and human metapneumovirus (score of 48.6). This top-ranking position of respiratory viruses may result, at least in part, from the effect of the COVID-19 pandemic. Apart from influenza virus [14,17] and RSV [14], these infectious entities were not prioritised in two studies that had been conducted in 2011 [14] and 2018 [17].

The single-criterion analysis that was carried out partially corrected some unexpectedly underestimated rankings obtained by the MCDA (e.g. poliomyelitis, measles, CMV congenital infection, virus-induced cancers, bacterial STIs or HIV infection, that represent serious challenges in terms of public health); certain entities that ranked low in the MCDA for which public health measures are already in place (such as surveillance, vaccination) were lifted in the top 10 using this approach. As shown in Table 4, criterion 10 that concerns unmet need in disease surveillance highlights the importance of neglected diseases such as some parasitic infections that are associated with a significant burden in vulnerable population, especially in overseas territories.

Finally, it is reassuring that most of the prioritised infectious entities defined in the present study are well covered by the European and national surveillance and alert systems [34,35]. As shown in Supplementary Table S3 for French surveillance structures coordinated by Santé publique France*,* all high-priority entities are subject to an NRL while only three low-priority entities (melioidosis, ectoparasitosis, and nocardiosis) are neither notifiable diseases nor subject to an NRL. Our list is also in agreement with the priority-for-research list published in 2023 by the French National Research Agency for Emerging Infectious Diseases (ANRS-MIE) [36].

## Conclusion

The present study is a further demonstration of the ability of the MDCA method recommended by the ECDC to prioritise infectious risks. The selection of entities and criteria, the choice of experts and raters, and the tool used for the survey enabled the analysis to be carried out in a relatively short period of time, allowing its periodic update. The degree of liberty offered by the MCDA approach also enabled to provide an analysis tailored to the French overseas territorial specificities, in which infectious risks differ greatly from that of mainland France. The priority list of infectious entities established in this study should help updating public health policies aimed at addressing existing risks and anticipating future ones.

## Supplementary Data

967000 Supplementary Material
