# Supplementary material for: Prioritisation of infectious diseases from a public health perspective: a multi-criteria decision analysis study, France, 2024
Source: Euro Surveill. 2024 Dec 12;29(50):2400074. doi: 10.2807/1560-7917.ES.2024.29.50.2400074 (PMC11650512; doi:10.2807/1560-7917.ES.2024.29.50.2400074)
Supplement: Supplementary Material [file 2400074_SupplementaryMaterial.pdf]

Supplementary materials for the article:

Prioritisation of infectious diseases from a public health perspective: a multi-criteria decision analysis study, France, 2024

This supplementary material is hosted by *Eurosurveillance* as supporting information alongside the article entitled "**Prioritisation of infectious diseases from a public health perspective: a multi-criteria decision analysis study, France, 2024"** on behalf of the authors, who remain responsible for the accuracy and appropriateness of the content. The same standards for ethics, copyright, attributions, and permissions as for the article apply. Supplements are not edited by *Eurosurveillance* and the journal is not responsible for the maintenance of any links or email addresses provided therein.

Supplementary Table S1: **p 2**

Synthetic presentation of the literature review

Supplementary Table S2: **p 3**

Distribution of 98 raters and number of rated entities according to medical specialty

Supplementary Table S3: **p 4**

Respective impact of the different criteria on the rating of the entities

Supplementary Table S4: **p 5**

Description of the incidence and case-fatality rates and of the existence of a notifiable disease (ND) system and a National Reference Laboratory (NRL) in France for the 94 ranked entities as well as Disease X

Supplementary Figure S1: **p 8**

Example of the tool "LimeSurvey": screen for rating Hepatitis E disease

Supplementary Figure S2: **p 9**

Inter-rater rating homogeneity analysis

Supplementary Figure S3: **p 10**

Consistency of responses between complete and partial raters

Supplementary Figure S4: **p 11**

Assessment of inter-rater dissensus (Disease X included)

Supplementary Figure S5: **p 12**

Distribution of the rating scores of the 95 entities and categorization in high-priority, low-priority, and non-priority

Supplementary Table S1: Synthetic presentation of the literature review. The prioritization lists were issued primarily by high income countries in Europe and North America. None has territories dispersed around the globe. Some of them used Multi-Criteria Decision Analysis (MCDA) to rank communicable disease risks.

Publication:<br>First author (year) - Country<br>DOI or reference	Methods:	Identified sample size:<br>N diseases/pathogens; N criteria<br>N participants for weighting; for rating
Klamer (2021) - Belgium<br>DOI : 10.1186/S12889-020-09566-9	**MCDA**	98 Communicable diseases / 18 weighted criteria<br>Weighting: n= 80; Rating: n =37
Otten (2019) - Canada<br>DOI :10.1016/j.mran.2019.100089	**MCDA**	43 vector-borne diseases / 10 weighted criteria<br>Number of participants unclear
Dahl (2015) - Sweden<br>DOI : 10.1371/journal.pone.0136353	Delphi method<br>(unknown n rounds)	106 pathogens / 10 unweighted criteria<br>Weighting: NA; Rating: n = 5
Brookes (2014a & b) – Australia<br>DOI: 10.1016/j.prevetmed.2013.10.014<br>DOI: 10.1016/j.prevetmed.2013.10.016	**MCDA**	30 pig diseases / 9 weighted criteria<br>Weighting: n = 50; Rating n=50
Economopoulou (2014) - European Union<br>DOI: 10.2807/1560-7917.es2014.19.15.20770	Delphi method<br>(2 rounds)	71 Communicable diseases / 2 unweighted criteria<br>Weighting: NA; Rating: n = 56
Cediel (2013) - Colombia<br>DOI: 10.1590/s1020-49892013000500002	Delphi method<br>(1 round)	32 zoonotic diseases / 12 weighted criteria<br>Weighting: n = 12; Rating: n = 12
Ng (2013-2012) - North America<br>DOI: 10.1371/journal. pone.0029752<br>DOI: 10.1371/ journal.pone.0048519<br>DOI: 10.1371/journal.pone.0072172	Questionnaire<br>(conjoint analysis)	62 zoonotic diseases / 21 weighted criteria<br>Public: n = 1539; Professionals: n = 1471
Cox (2013, 2012) - Canada<br>DOI: 10.1371/journal.pone.0041590<br>DOI: 10.1371/journal.pone.0068338	**MCDA**	9 Communicable diseases / 40 weighted criteria<br>Weighting: n = 64; Rating: n = 47
Humblet (2012) - Belgium<br>DOI: 10.3201/eid1804.111151	**MCDA**	100 zoonotic diseases / 57 weighted criteria<br>Weighting: n = 40; Rating procedure not available
Balabanova (2011) - Germany<br>DOI: 10.1371/journal.pone.0025691	Delphi method<br>(1 round)	127 pathogens / 10 weighted criteria<br>Weighting: n = 86; Rating: n = 20
Capek (2010) - France<br>Reference[1]	Expert opinion	37 non-food borne zoonoses / 10 unweighted criteria<br>Weighting: NA; Rating: n = 16
Havelaar (2010) - The Netherlands<br>DOI: 10.1371/journal.pone.0013965	**MCDA**	86 zoonotic diseases / 7 weighted criteria<br>Weighting: n = 29; Rating: unclear size of the panel.
Cardoen (2009) - Belgium<br>DOI: 10.1089/fpd.2009.0291	**MCDA**	51 zoonotic pathogens / 5 weighted criteria.<br>Weighting: n = 7; Rating: n = 35
Krause (2008) - Germany<br>DOI: 10.1038/embor.2008.76	Delphi method<br>(1 round)	85 pathogens / 12 weighted criteria<br>Weighting: n = 11; Rating: n = 11
Doherty (2006) - Canada<br>PMID: 17076030	Expert opinion	48 Communicable diseases / 10 unweighted criteria<br>Weighting: NA; Rating: n = 6
Who (2002) - 7 countries of South-East Europe<br>http://apps.who.int/iris/handle/10665/107469	Delphi method<br>(1 round)	53 Communicable diseases / 8 unweighted criteria<br>Weighting: NA; Rating: n = 24
Doherty (2000) - Canada<br>DOI: 10.1155/2000/134624	Expert opinion	43 Communicable diseases / 10 unweighted criteria<br>Weighting: NA; Rating: n = 6
Carter (1991) - Canada<br>DOI :10.1155/1991/346135	Expert opinion	60 Communicable diseases / 12 unweighted criteria<br>Weighting: NA; Rating: n = 6

NA: not applicable due to lack of weighting process.

[1] https://www.santepubliquefrance.fr/docs/definition-des-priorites-dans-le-domaine-des-zoonoses-non-alimentaires-2008-2009

Supplementary Table S2: Distribution of the 98 raters and number of rated entities according to medical specialty.

Specialty	Participants [a] (n1) among proposed experts (n2)		Rated entities	
	n1/n2	%	n	%
Infectious diseases	22/24	91.7	1216	26
Emergency medicine	19/34	55.9	836	18
Public health	11/13	84.6	684	14
Intensive care medicine	12/25	48.0	532	11
Paediatrics	11/18	61.1	513	11
General medicine	11/21	52.4	475	10
Geriatrics	6/18	33.3	304	6
Occupational health	6/16	37.5	190	4

[a] Fourteen learned societies were stakeholders of the study and proposed 169 representative participants (participation rate 58%). The experience of participants in the specialty was distributed as follows: less than 10 years for 21 participants (21.4%); between 10 and 20 years for 36 participants (36.7%); between 20 and 30 years for 17 participants (17.3%); and over 30 for 24 participants (24.5%).

Supplementary Table S3: Description of the incidence and case-fatality rates and of the existence of a notifiable disease (ND) system and a National Reference Laboratory (NRL) in France for the 94 ranked entities as well as Disease X (the background for the high-priority, low priority, and non-priority groups of entities are pink, yellow, and green, respectively).

Entities	Incidence rate	Case-fatality rate	ND	NRL
**E0-** Disease X	Minimal	Simulated from Minimal to High	NA	NA
**E1-** Viral haemorrhagic fevers	Minimal	High	YES	Viral haemorrhagic fevers
**E2-** Acute respiratory infections due to viruses other than influenza, emerging coronaviruses, RSV and hMPV	High[1]	Moderate	YES. for some	Respiratory virus including influenza virus and SARS-CoV-2
**E3-** Mosquito-borne arboviroses	High[2]	Low	YES. for some	Arbovirus
**E4-** Influenza virus infections with zoonotic potential	Minimal	High	NO	Respiratory virus including influenza virus and SARS-CoV-2
**E5-** Seasonal influenza A and B	High	Low	NO	Respiratory virus including influenza virus and SARS-CoV-2
**E6-** Emerging coronavirus infections (Severe Acute Respiratory Syndrome, Middle-East Respiratory Syndrome, COVID-19)	Low	Low	YES. for some	Respiratory virus including influenza virus and SARS-CoV-2
**E7-** Respiratory syncytial virus (RSV) and human metapneumovirus (hMPV) respiratory infections	High	Low	NO	Respiratory virus including influenza virus and SARS-CoV-2
**E8-** Creutzfeldt-Jakob disease and other human transmissible spongiform encephalopathies (TSEs)	Minimal	High	YES	Non-conventional transmissible agents
**E9-** Systemic infections due to multi-resistant bacteria to antibiotics	Low	Moderate	NO	Antibiotic resistance
**E10-** Infections due to emerging highly-resistant bacteria to antibiotics	Minimal	Minimal	NO	Antibiotic resistance
**E11-** Invasive infections due to *Neisseria meningitidis*	Minimal	High	YES	Meningococci and *Haemophilus influenzae*
**E12-** Rabies	Minimal	High	YES	Rabies
**E13-** Tuberculosis due to antibiotic-susceptible strains	Low	Moderate	YES. for some	Mycobacteria and resistance to anti-tuberculosis drugs
**E14-** Invasive pneumococcal disease	Low	High	NO	Pneumococcus
**E15-** Plague	Minimal	High	YES	Plague and others yersinioses
**E16-** Invasive yeast and filamentous fungal infections (*Candida*, *Aspergillus…)*	Moderate	Moderate	NO	Invasive and antifungal fungal mycoses
**E17-** Severe infections due to *Enterobacterales*	High	Low	NO	*E. coli*. shigella. salmonella
**E18-** Rotavirus gastroenteritis	High	Minimal	NO	Gastroenteritis virus
**E19-** Multi-drug tuberculosis	Low	Low	YES. for some	Mycobacteria and resistance to anti-tuberculosis drugs
**E20-** Tetanus	Minimal	High	YES	Anaerobic bacteria and botulism
**E21-** Viral gastroenteritis excluding rotavirus	High	Minimal	NO	Gastroenteritis virus
**E22-** Listeriosis	Low	High[3]	YES	*Listeria*
**E23-** Invasive infections due to *Staphylococcus aureus*	High	Low	NO	Staphylococci
**E24-** Invasive tropical mycoses	Minimal	High	NO	Invasive and antifungal fungal mycoses
**E25-** Melioidosis	Minimal	High	NO	NONE
**E26-** Measles	Minimal	Minimal	YES	Measles. rubella and mumps virus
**E27-** Cutaneous infections of aquatic origin	Minimal	High	NO	Vibrios and cholera
**E28-** Human immunodeficiency virus (HIV) infection	Low	Low	YES	Human immunodeficiency virus
**E29-** Food-borne gastroenteritis/food poisoning	High	Minimal	YES	*Campylobacter* and *Helicobacter* *E. coli*. shigella. salmonella

**E30-** Severe viral infections in immunocompromised patients	Low	Moderate	NO	Herpes virus
**E31-** Ectoparasitoses including scabies, pediculosis and bed bug infestation	Moderate	Minimal	NO	NONE
**E32-** Bacterial sexually-transmitted infections	Moderate	Minimal[4]	NO	Bacterial sexually transmitted infections
**E33-** Cancers and other diseases caused by human papillomaviruses	Low	Low	NO	Human papillomavirus
**E34-** Orthopoxvirus infections including smallpox and Mpox	Minimal	Minimal	YES	Orthopoxvirus including smallpox and Mpox
**E35-** Invasive infections due to *Streptococcus pyogenes* and other invasive streptococci (*S. suis. S. dysgalactiae*)	Moderate	Low	NO	Streptococci
**E36-** Enteroviruses excluding poliomyelitis	High	Minimal	NO	Enterovirus and parechovirus
**E37-** Botulism	Minimal	Moderate	YES	Anaerobic bacteria and botulism
**E38-** Legionellosis	Minimal	Moderate	YES	*Legionella*
**E39-** Tick-borne encephalitis	Minimal	Low	YES	Arbovirus
**E40-** Haemolytic uremic syndrome	Minimal	Low	NO	*Escherichia coli*. shigella. salmonella
**E41-** Diphtheria	Minimal	Moderate	YES	*Corynebacteria* of the dipthteriae complex
**E42-** *Clostridioides difficile* infections	Moderate	Minimal	NO	Anaerobic bacteria and botulism
**E43-** Malaria	Low	Low	YES *Autochthonous and imported malaria in the DOM*	Malaria
**E44-** Nocardiosis	Low	Moderate	NO	NONE
**E45-** Cholera	Minimal	Minimal	YES	Vibrios and cholera
**E46-** Infections caused by hypervirulent clonal strains of *Klebsiella pneumoniae*	Minimal	Minimal	NO	*E. coli*. shigella. salmonella
**E47-** Invasive infections due to coagulase-negative staphylococci	High	Minimal	NO	Staphylococci
**E48-** Invasive infections due to *Haemophilus influenzae* serotype b	Minimal	Moderate	NO	Meningococci and *Haemophilus influenzae*
**E49-** Poliomyelitis	Minimal	Minimal	YES	Enterovirus and parechovirus
**E50-** Diseases induced by *Helicobacter pylori*	High	Minimal	NO	*Campylobacter* and *Helicobacter*
**E51-** Cancers induced by and severe infections due to Human Herpes Virus (HHV) type 8	Minimal	Low	NO	Herpes virus
**E52-** Cancers induced by and severe infections with Epstein-Barr virus (EBV)	Minimal	Low	NO	Herpes virus
**E53-** Diseases induced by human T-lymphotropic virus (HTLV) types 1 & 2	Minimal	Minimal	NO	NONE
**E54-** Congenital cytomegalovirus (CMV) infection	Low	Minimal	NO	Herpes virus
**E55-** Gastroenteritis and parasitic enterocolitis	Moderate	Minimal	NO	Cryptosporidiosis. microsporidia and other digestive protozooses
**E56-** Hepatitis B / Hepatitis D	Low	Low	YES *Symptomatic acute HBV infection*	Hepatitis B. C and Delta virus
**E57-** Anthrax	Minimal	Low	YES	Anthrax
**E58-**Systemic enterococcal infections (*Enterococcus faecalis*. *E. faecium*)	Low	Low	NO	NONE
**E59-** Hepatitis C	Low	Minimal	NO	Hepatitis B. C and Delta virus
**E60-** Mycobacteriosis (excluding tuberculosis and leprosy)	Low	Minimal	YES. for some	Mycobacteria and resistance to anti-tuberculosis drugs
**E61-** Leprosy	Minimal	Minimal	NO	Mycobacteria and resistance to anti-tuberculosis drugs
**E62-** Leptospirosis	Low	Low	YES	Leptospirosis
**E63-** Chagas disease	Minimal	Minimal	NO	NONE
**E64-** Pneumocystis pneumonia	Low	Low	NO	Invasive and antifungal fungal mycoses
**E65-** Whooping cough	Low [5]	Minimal	NO	Whooping cough and other bordetelloses
**E66-** Atypical pneumonia	Moderate	Minimal	NO	NONE
**E67-** Congenital rubella	Minimal	Minimal	YES	Measles. rubella and mumps virus
**E68-** Haemorrhagic fever with renal syndrome (Puumala hantavirus)	Minimal	Minimal	NO	Hantavirus
**E69-** Urogenital/intestinal schistosomiasis	Minimal	Minimal	YES *Autochthonous urogenital schistosomiasis*	NONE
**E70-** Cutaneous or visceral leishmaniases	Low[6]	Minimal	NO	Leishmaniases

**E71-** Hepatitis E	Minimal	Minimal	NO	Enteric hepatitis viruses (hepatitis A and E)
**E72**- Lyme disease	Low	Minimal	NO	*Borrelia*
**E73-** Typhoid and paratyphoid fevers	Minimal	Low	YES	*Escherichia coli*. shigella. salmonella
**E74-** Cystic and alveolar echinococcoses	Minimal	Low	NO	Echinococcoses
**E75-** Parvovirus B19 infection	Moderate	Minimal	NO	NONE
**E76-** Varicella zoster virus infection	Moderate	Minimal	NO	Herpes virus
**E77-** Congenital toxoplasmosis	Minimal	Minimal	NO	Toxoplasmosis
**E78-** Severe herpes simplex virus types 1/2 infections	Minimal	Low	NO	Herpes virus
**E79-** Q fever	Minimal	Low	NO	*Rickettsia. Coxiella. Bartonella*
**E80-** Rickettsioses	Minimal	Low	YES. for some	*Rickettsia. Coxiella. Bartonella*
**E81-** Systemic *Streptococcus agalactiae* infections	Low[7]	Minimal	NO	Streptococci
**E82-** Hepatitis A	Minimal	Minimal	YES	Enteric hepatitis viruses (hepatitis A and E)
**E83-** Dermatophytoses (*Microsporum* and *Trichophyton*)	Moderate	Minimal	NO	NONE
**E84-** Intestinal nematodiases	Moderate	Minimal	NO	NONE
**E85-** Anaplasmosis and other tick-borne bacterial infections (*Ehrlichia*)	Minimal	Minimal	NO	NONE
**E86-** Whipple's disease	Minimal	Minimal	NO	NONE
**E87-** Filariases. cutaneous. visceral *larva migrans*	Low	Minimal	NO	NONE
**E88-** Tularemia	Minimal	Minimal	YES	*Francisella tularensis*
**E89-** Mumps	Minimal	Minimal	NO	Measles. rubella and mumps virus
**E90-** Bartonellosis	Minimal	Low	NO	*Rickettsia. Coxiella. Bartonella*
**E91-** Brucellosis	Minimal	Minimal	YES	*Brucella*
**E92-** Bacterial inoculation diseases (red mullet, haverhilliosis)	Minimal	Low	NO	NONE
**E93-** Distomatoses	Minimal	Minimal	NO	NONE
**E94-** Pasteurellosis	Minimal	Minimal	NO	NONE

NA: not applicable

1 Function of virus, age, and presence of risk factors.

2 For dengue fever or Chikungunya in the West Indies and Reunion Island.

3 Non-maternal-neonatal forms.

4 Excluding congenital syphilis.

5 Data for patients < 12 months hospitalised in the RENACOQ network.

6 Cutaneous leishmaniasis in French Guiana ≈ 67 cases/100,000 inhabitants.

7 For the French overseas territories.

Supplementary Table S4: Respective impact of the different criteria on the rating of the 94 entities and the 14 high-priority entities, excluding Disease X.

Criterion	94 entities (%)	14 high-priority entities (%)
**C1:** Potential for the emergence and spread of the disease or pathogen	12.9	14.5
**C2**: Annual incidence (number of cases / 100,000 inhabitants)	6.3	5.3
**C3:** Case-fatality rate (number of fatal cases / number of cases)	11.5	17.7
**C4**: Individual impact on the patient	20.4	14.7
**C5:** Societal impact	9.1	10.2
**C6**: Impact on the healthcare system	9.1	12.8
**C7:** Impact on socially vulnerable populations	12.3	9.7
**C8:** Unmet need for prevention	7.0	4.9
**C9:** Unmet need for curative treatment	8.4	8.6
**C10:** Unmet need in disease surveillance in mainland and overseas France	3.1	1.7

Supplementary Figure S1: Example of the tool "LimeSurvey": the screen for Hepatitis E disease. It was displayed with its initial numbering M53 (for didactic purpose in the article, each entity was renumbered according to its ranking, e.g. E71 for hepatitis E disease).

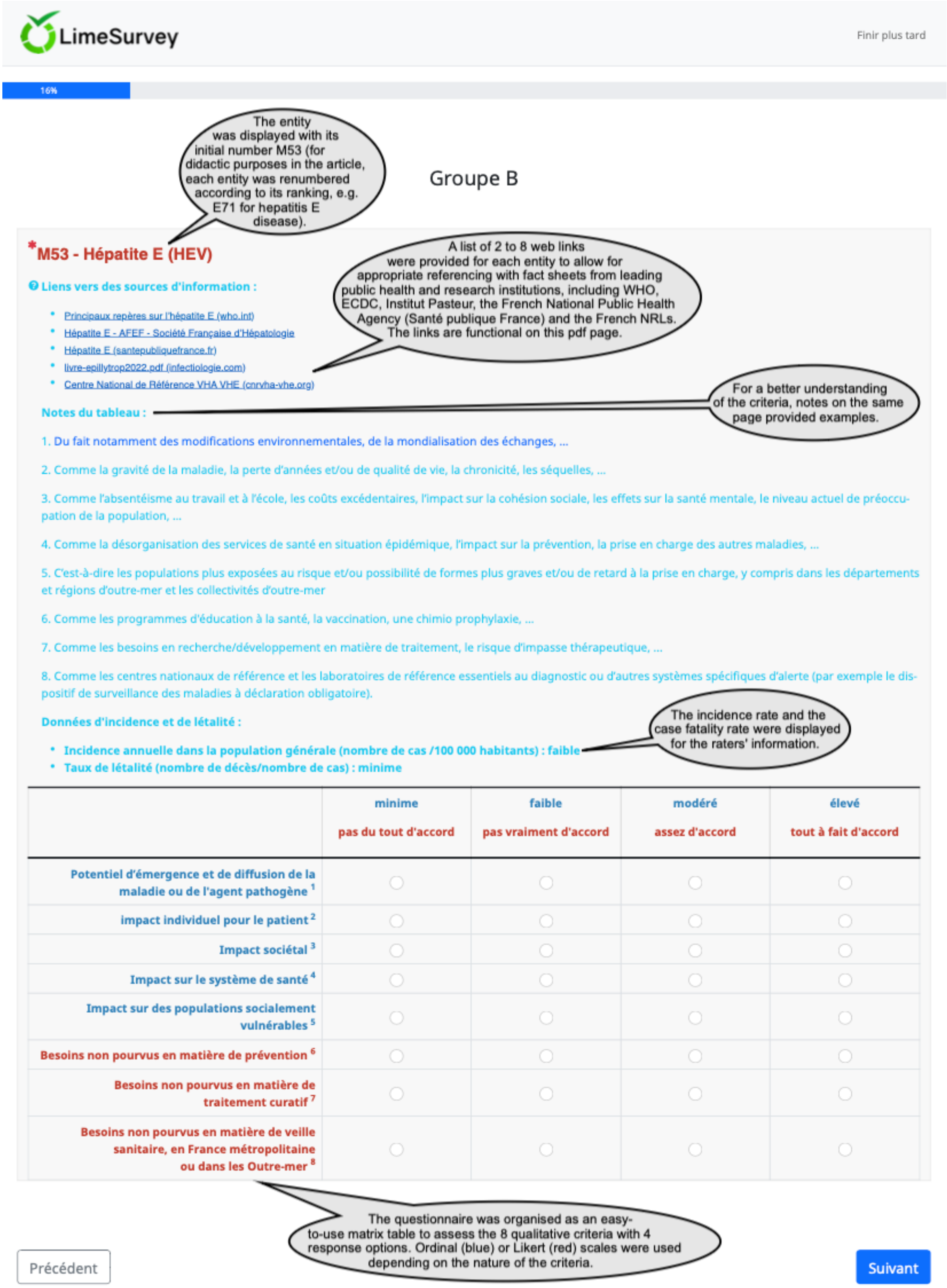

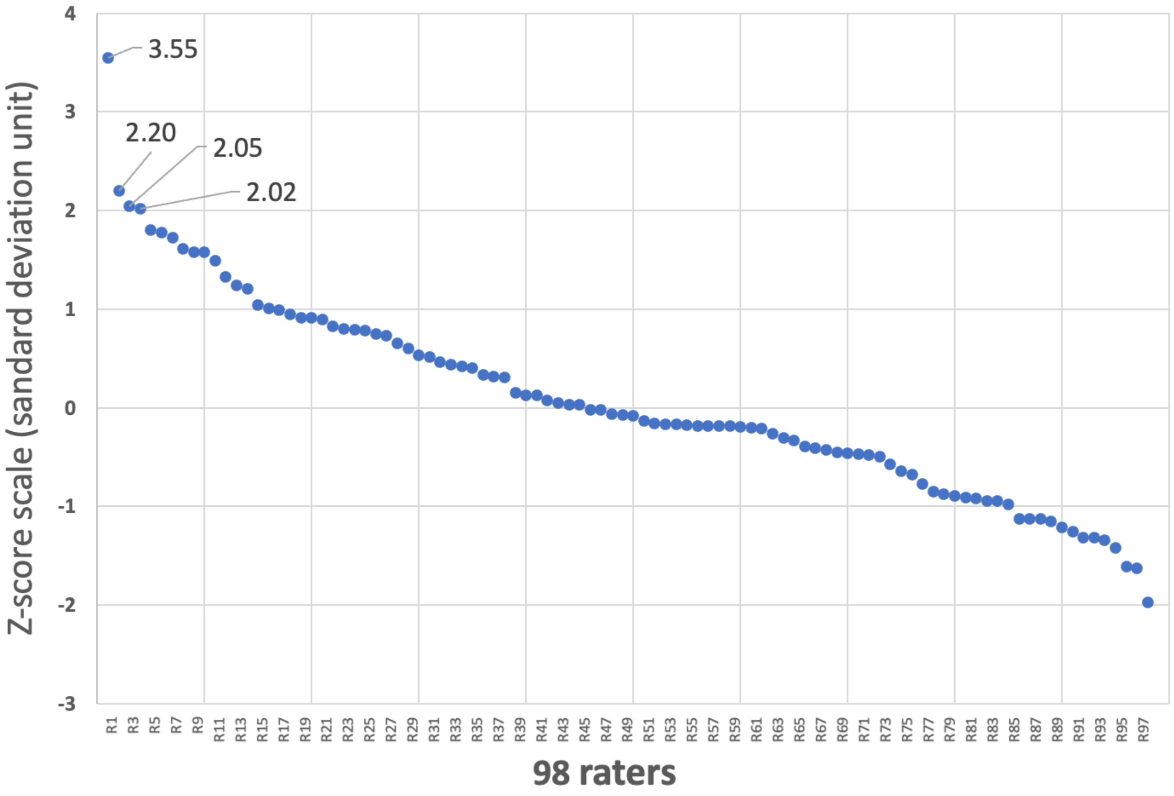

Supplementary Figure S2: Inter-rater rating homogeneity analysis. Inter-rater rating homogeneity was analysed by ranking the average score of the 98 raters on a Z-score scale and identifying values that differed by ± 2 ds.

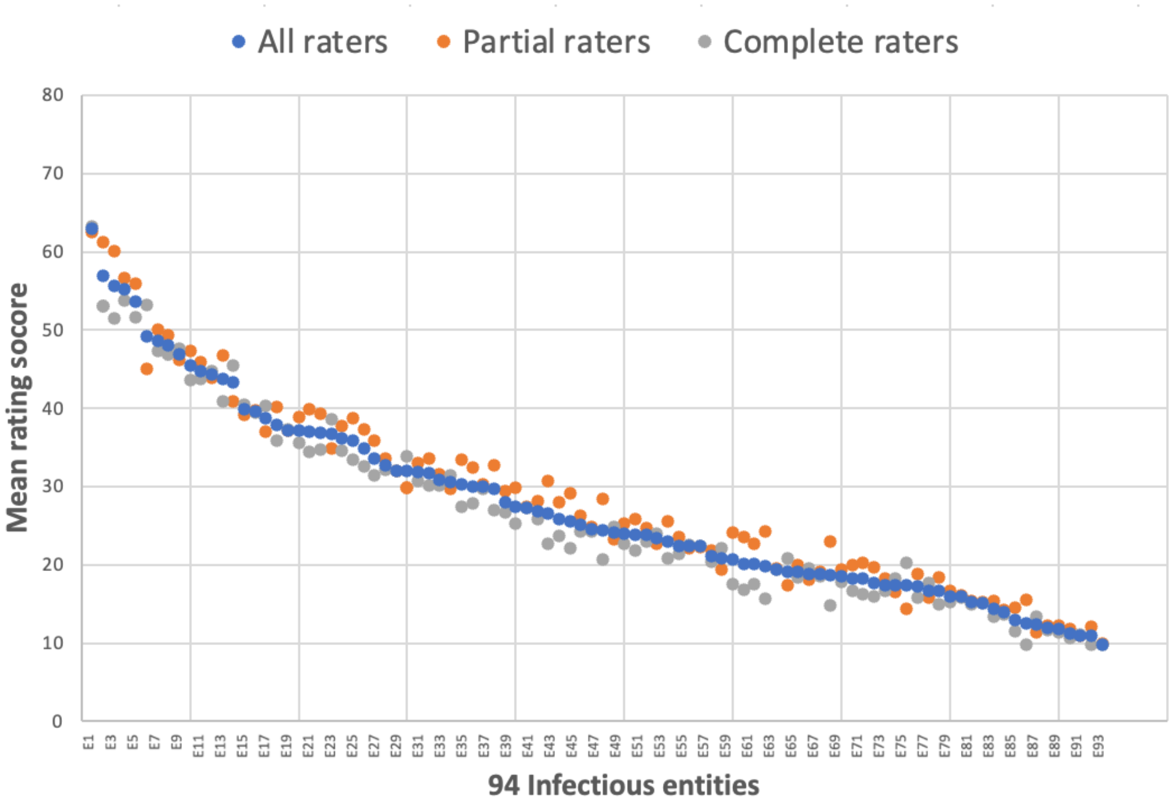

Supplementary Figure S3: Consistency of responses between complete and partial raters

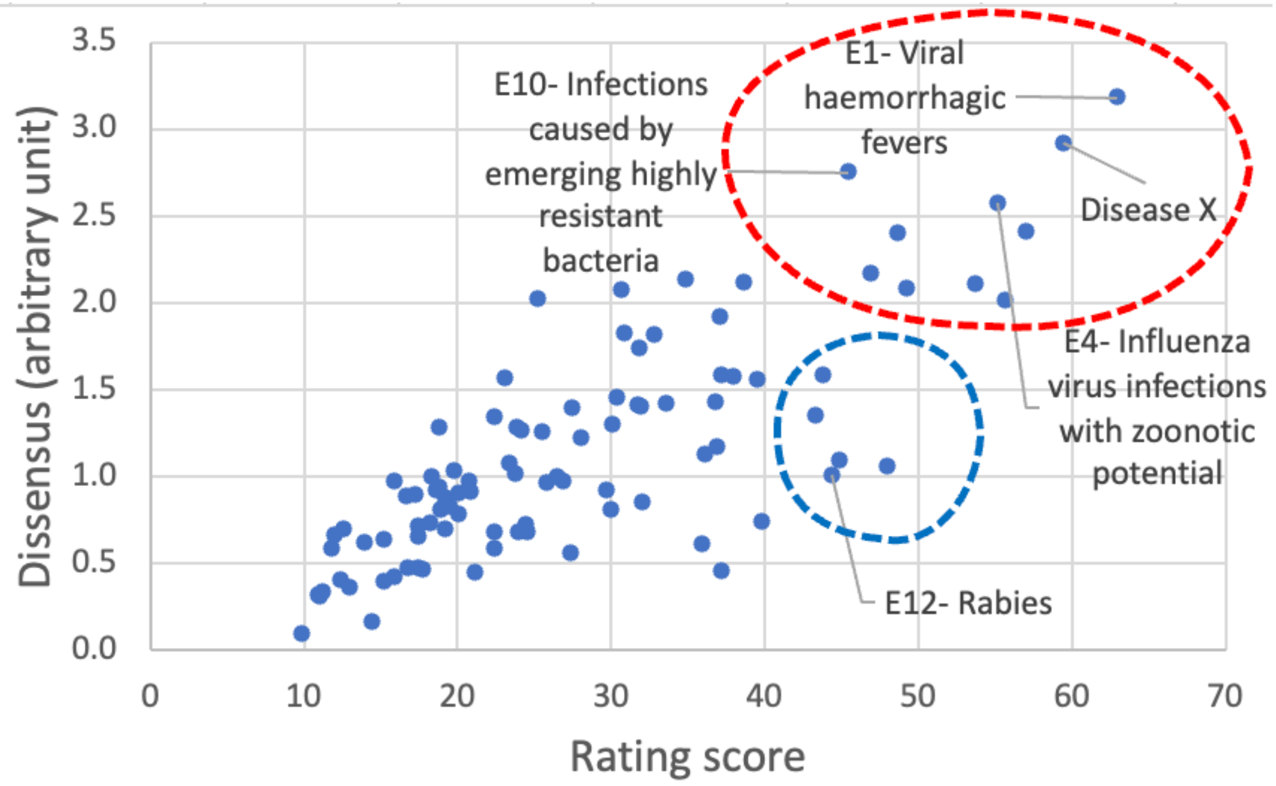

Supplementary Figure S4: Assessment of inter-rater dissensus (Disease X included). Entities in the upper-right quarter of the diagram require particular attention as they are associated with high dissensus (ordinate) and high scores (abscissa); by contrast, entities in the middle of the diagram do not seem to generate strong dissensus despite relatively high scores.

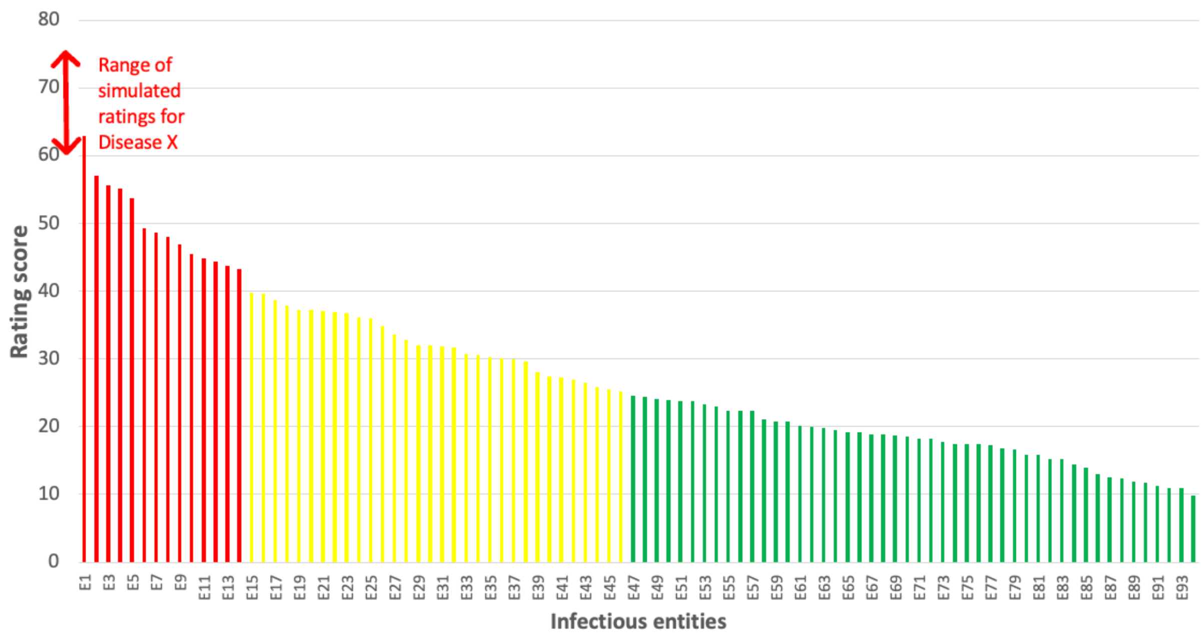

Supplementary Figure S5: Distribution of the rating scores of the 94 entities as well as Disease X and categorisation according to three priority levels. The entities with a rating score higher than 40 were considered as high-priority (red), those with a rating score between 25 and 40 were considered as low-priority (yellow), and those under 25 were categorised as non-priority (green). The simulated ratings for E0—Disease X range from 59.4 to 75.4.
